# Understanding the decision making process of selection of medicines in the private sector in South Africa – lessons for low-middle income countries

**DOI:** 10.1186/s40545-020-00223-5

**Published:** 2020-05-21

**Authors:** Velisha Ann Perumal-Pillay, Fatima Suleman

**Affiliations:** Discipline of Pharmaceutical Sciences, School of Health Sciences, University of KwaZulu-Natal, Durban, South Africa

**Keywords:** Essential medicines, Private sector healthcare, Selection of medicines, Formulary development and management, South Africa

## Abstract

**Background:**

The affordability of essential medicines is a challenge in achieving Universal Health Coverage (UHC). One of The Lancet Commission’s recommendations on financing of essential medicines is to ensure governments and national health systems include essential medicines in the benefit packages provided by public and private healthcare sectors. Currently in South Africa (SA), there is a dearth of information on the processes for medicines selection for private sector medical scheme formularies. This study aimed to improve the understanding of how formulary managers of selected medical schemes made decisions for the selection of medicines for their formularies. This paper described their opinions obtained from in-depth interviews.

**Methods:**

Qualitative in-depth interviews were conducted with 10 individuals from 7 private sector medical schemes and administrators in SA. All participants interviewed were involved in formulary development and management. Interviews were conducted from June 2013 – January 2015. Interviews were guided by a discussion guide and audio recorded. Recorded interviews were transcribed verbatim. Transcripts were coded by the first author, corroborated by the second author, reconciled, and imported into NVIVO for data analysis.

**Results:**

Schemes and administrators had similar formulary decision making and management committees in place (viz. Clinical and Therapeutics committees). The process of and criteria for medicines selection and evidence based review of formularies were also similar. Selection of medicines was inherent in the formulary review process. Medicine price was important in the decision taken to list medicines. Most schemes expressed a difficulty with lack of information to support pharmacoeconomic evaluations of medicines for inclusion on the formulary. This together with the basic monitoring of use of medicines by patients for most schemes left room for improvement in the decision making process for those schemes.

**Conclusions:**

This is one of the first studies in SA describing interviews with private sector medical scheme Formulary managers. It contributes to an increased understanding of how decisions are taken to include/exclude medicines on private sector medical scheme formularies. It provides insight into the medicine selection and review processes, including processes on monitoring and evaluation of medicines use by the private sector which serve as lessons for Low-Middle income countries moving towards UHC.

## Background

In the years following the first democratic election in South Africa (SA) in 1994, the country’s healthcare system was subjected to numerous transformations to improve the provision of equitable access to healthcare for all. However this still remains a challenge. The healthcare system still operates in parallel a public sector (government funded) and a private sector (insured by medical schemes). The World Health Organization (WHO) Global Health expenditure database (2014) showed government’s expenditure on healthcare to be 48% of total expenditures in the public sector and 52% in the private sector [1]. Despite this almost equivalent spending, only 17% of the South African population are insured by private medical schemes [2]. Approximately 40% of healthcare in SA is financed by government tax revenue; approximately 45% is funded by private medical schemes and approximately 14% is funded by out-of-pocket payments [3]. When out-of-pocket expenditures are included, an estimated 28–38% of the population are accessing private healthcare services indicating an increase in the number of people accessing the private sector for healthcare [2].

Private healthcare funding in SA is primarily provided by medical schemes. Medical schemes are not-for-profit organizations, mostly privately funded but also receive a tax subsidy. Some medical schemes manage their own day-to-day issues such as membership queries, claims processing, management of clinical issues and costs. These schemes are referred to as self-administered whilst some schemes contract an Administrator to handle such issues. Some Administrators provide managed care services but there are also managed care organisations that provide a specific service to measure and monitor services provided to medical scheme beneficiaries. Managed care strategies aim to balance healthcare requirements and costs [2]. Medical schemes, Administrators and Managed Care Organisations register with the Council for Medical Schemes (CMS), a legislative body established by the Medical Schemes Act (131 of 1998) tasked with regulating medical schemes in South Africa [4]. The main forms of medical schemes are (i) open (available to all consumers) and (ii) restricted (membership is limited to certain company employees) schemes. Both types may offer various products known as benefit options. These benefit options differ in design both between medical schemes and within medical schemes and range from basic packages to very comprehensive packages and are tailored by each scheme [5]. Every benefit option is approved by and registered with the CMS to ensure their design is aligned with legal requirements of the Medical Schemes Act (131 of 1998). A common feature in all benefit options is the Prescribed Minimum Benefits (PMBs). According to the Medical Schemes Act (131 of 1998) [6], PMBs are the mandatory minimum level of benefits that all options are required to provide and are described as follows [4]:

“a set of defined benefits to ensure that all medical scheme members have access to certain minimum health services, regardless of the benefit option they have selected. The aim is to provide people with continuous care to improve their health and well-being and to make healthcare more affordable”.

The PMB package comprises [4]:

- 270 diagnosis-treatment pairs
- Emergency treatment
- 25 chronic disease conditions (defined in a chronic disease list (CDL))

The Medical Schemes Act stipulates that medical schemes must pay the full costs for the diagnosis, treatment and care of these PMBs with no co-payments or deductibles. This statement is perceived as problematic as there is no national guideline of service provider charges. The Act therefore makes provision for the use of managed care techniques to contain the impact of PMBs on affordability [6]. These techniques include:

- Formularies or medicine lists
- Treatment protocols with clinical entry criteria
- Treatment algorithms
- Benefit confirmation for procedures
- Designated service providers

Although the intention of PMBs was to improve healthcare and make it affordable, the current PMBs structure is perceived as “unsustainable” as it creates a high-base cost of cover; prevents the medical schemes from designing their own affordable package of benefits for consumers; and is seen as unsuitable for the current healthcare reform environment in SA [5] given the implementation of a National Health Insurance (NHI) financing scheme as the country moves towards Universal Health Coverage (UHC).

The WHO describes UHC as follows [7]:

“UHC means that all people and communities can use the promotive, preventive, curative, rehabilitative and palliative health services they need, of sufficient quality to be effective, while also ensuring that the use of these services does not expose the user to financial hardship”.

The attainment of UHC should ensure equity in access to quality health services to all without inflicting financial harm. The affordability of essential medicines remains a challenge in health systems aiming to achieve UHC. Globally, healthcare systems are experiencing difficulty harmonizing expanding and maintaining appropriate pharmaceutical benefits coverage and ensuring quality healthcare, efficient spending and reduced out-of-pocket payments. However, the Lancet Commissions’ on essential medicines proposes that UHC can be achieved by policies that support essential medicines which must support health services delivered through mixed systems including both the public and private sectors [8].

Essential medicines should be covered in a medical scheme’s PMBs. One of the managed care interventions for medical schemes to contain costs with PMBs is the use of formularies. A formulary is a list of prescription drugs determined to be clinically appropriate and cost-effective and that are approved for use and covered by a medical scheme [9]. Medical schemes will then reimburse items listed on their formularies up to a certain price [10].

The careful selection of medicines for a formulary is a cost-containment tool in itself. Little is known about how medical schemes in SA develop their formularies. There is a dearth of information on the processes for medicines selection for private sector formularies. This study aimed to improve the understanding of how medical scheme formulary managers made decisions for the selection of medicines, including essential medicines, for their schemes’ formularies. This paper describes their opinions as obtained from in-depth interviews.

## Methods

### Sample selection

The sample was selected from the list of companies (medical schemes, administrators and managed care organisations) registered with the Council for Medical Schemes (CMS). The CMS is a legislative body established by the Medical Schemes Act (131 of 1998) to regulate private healthcare financing through medical schemes [4]. The sample was selected based on information from the 2012/3 CMS Annual Report [11]. Participants were purposively selected based on their membership numbers in the hopes that the companies with the most number of scheme beneficiaries would be included in the study sample. Invitations to participate were sent to the 10 companies with the highest numbers of beneficiaries. However, only 8 companies responded of which 1 declined the invitation to participate. The sample then comprised 7 companies and included 3 large companies who were administrators of medical schemes to more than three quarters of medical scheme beneficiaries at that time. Thus, the sample was representative of the majority of the population covered by medical schemes. Data saturation was achieved during the interview process.

### Development of the instrument

The interview guide used in this study was adapted from the American Society of Health-system Pharmacists formulary questionnaire for formulary management [12]. The first draft of the interview guide was piloted with a member of the Benefit and Risk Division at the Board of Healthcare Funders of Southern Africa, which is a representative body to the health care funding industry. Members of the Board of Healthcare Funders include medical schemes, administrator organisations, and managed care organisations in Southern African countries, including South Africa [13]. The interview guide was amended as recommended during this pilot interview. The interview guide comprised open ended questions concerning formulary decision making committees, their membership, roles and responsibilities; management policies, the committees’ process of selection of medicines and how the committee reviewed and maintained the formulary as well as monitored the use of medicines by patients.

### Data collection

The interviews were conducted from June 2013 – January 2015. Interviews were carried out by the researcher who had no previous knowledge of private sector formularies and formulary development and management thereby eliminating possible bias and influence from the researcher.

### Data processing and analysis

All participants were interviewed in English as all were fluent in the language. Qualitative in-depth interviews were conducted with 10 individuals from 7 private sector companies who were either medical scheme administrators (*n* = 5), or self-administered medical schemes (*n* = 2). All participants interviewed were top-level administrators involved in formulary development and management for their particular company. A discussion guide guided interviews. Interviews were conducted face-to-face, lasted between 30 and 40 min and were audio tape-recorded. Recordings were then transcribed verbatim. The resultant transcripts were coded with NVIVO (version 10) software for qualitative analysis. A thematic analysis of data was conducted, based on grounded theory. Data was coded and classified then re-classified into sub-codes to make sure no new themes emerged. The data and codes were corroborated by the second author, then discussed by both authors for agreement on codes and themes.

### Ethics statement

The study was granted ethical clearance by the University of KwaZulu-Natal Human and Social Sciences Research Ethics Committee (HSS/0154/013). Consent to conduct the interviews with selected company formulary managers was obtained by informed consent forms which were signed prior to participation.

## Results

### Response rates and description of sample

Only 8 out of the 10 companies originally invited to participate, responded and only 7 agreed to participate in the study, namely 2 self-administered medical schemes and 5 medical scheme administrators, of which 4 also provided managed care services. This sample was representative of the majority of beneficiaries of medical schemes in SA as the sample included 3 large administrators of medical schemes who collectively contracted more than three quarters of medical scheme beneficiaries, and also included one of the largest self-administered schemes, at the time of the study. Furthermore, the four most densely populated provinces in SA were Gauteng (23.7%), KwaZulu-Natal (19.8%), Eastern Cape (12.7%) and Western Cape (11.2%) representing 67.4% of the total population [14], which were also the areas of concentration of most medical scheme beneficiaries, collectively representing 75% of medical scheme beneficiaries in 2012 [11]. All participants were involved with formulary development and management. At two companies more than one formulary manager participated, hence the sample comprised 10 participants (see Table 1).

Key themes derived from the interviews were: 1. Formulary decision making committees; 2. Medicine selection for formularies; 3. Monitoring and evaluation of outcomes; and 4. Challenges. These are discussed separately in the sections to follow.

### Formulary decision-making committees

This section describes the sub-themes concerned with the types of decision-making committees; their membership, roles and responsibilities, conflict of interest and how the committees developed and managed its policies.

#### Types of decision making committees, Membership, roles and responsibilities and conflict of interest declarations

Participants were asked to describe the committees responsible for formulary decisions and the membership thereof. Responses were unanimous that formulary decision making was indeed a “team effort” involving mainly a Clinical team and a Therapeutics team.

##### Formulary decision making committees; membership and roles and responsibilities

*a)Clinical Committee:* The Clinical committee fell within the medicine management division, which comprised experts in different disease conditions or specialist areas, including pharmacists. These individuals kept abreast with research developments, new entries to the market and existing medicines with new indications. They used evidence based medicine principles to gather information, including evidence on cost effectiveness and pharmacoeconomic studies were available, to compile a draft funding decision document for presentation to the Therapeutics Committee.

*b)Therapeutics committee:* The Therapeutics committee made a decision for inclusion on the formulary after careful consideration of the draft document submitted by the Clinical committee. Membership of the Therapeutics committees might comprise the following individuals: Representation from hospital benefit management; from prescribed minimum benefits; from medicine management; from medical advisory services; business risk management; managers from respective clinical divisions, drug utilisation review pharmacists; and external consultants. In most cases, the external stakeholders sat on the therapeutics committee as opposed to consultation on a need basis, which was the case with one medical scheme. This was to keep independence in their decision making process and the membership around it. External consultants might include academics with strong evidence based decision-making experience, medical doctors, and specialists. Consultants generally did not have a stake in the business. One scheme had a panel of expert consultants if the need for those expert opinions arose. The Therapeutics committee was responsible for the clinical decision-making.

Other committees that might be consulted in the decision making process are described below:

“if on the basis of price and clinical value, we need to evaluate whether the product gets listed, then we send that to the health economics unit”

Another participant simply stated they also consulted their drug reimbursement committee for funding decisions.

“The formulary decisions are made by the health policy unit so that would be my team plus from operations the coding team as well as another pharmacist who is in charge of the price file”.

##### Conflict of interest

The discussion surrounding conflicts of interest revealed that most companies felt they were not conflicted in any way despite having external consultants on their Therapeutics committees. Participants directed their comments towards their interactions with the pharmaceutical industry saying that there was no representation from pharmaceutical industries on their Therapeutics committees, hence there was no conflict.

Their responses included:

“We don’t have any involvement with pharmaceutical companies, we do meet with them, but we don’t have conflicts of interest”.

“Our D&T (drugs and therapeutics) committee has a standing agenda item where we would declare our conflicts of interest … any funding for conferences or whatever may need to be declared by various management”.

“In every meeting we’ve got declaration of interests which are signed by members and depending on the nature of any conflict, the chairman will decide whether the person is excluded from the discussion around a particular decision or not”.

#### The development and management of committee policies

Companies viewed their Therapeutics committees as multi-disciplinary with wide representation from experts in various fields and felt they were competent to develop and manage their own policies with minimal consultations for the most part. However, big business decisions would require wider consultation. Administrators responded that they would need to consult with their contracted schemes for big business decisions. Administrators and schemes would also consult their risk management teams, clinical innovations teams and other committees involved in benefit design.

The review of committee policies varied amongst companies but the one common response was “policies were reviewed annually or on an ad-hoc basis as the need arose”. Other responses included:

“Policies are revised intermittently as and when new products are launched and when pricing dynamics change”.

“If the pricing dynamic changes we would need to review our formulary status … the other thing would be if treatment guidelines are published and they differ from our policy we need to consider our policy”.

“Whenever there’s a new class of medicine that needs to be added, new technology medicine”

### The selection of medicines

This section describes the primary medicines selection process and criteria for selection. Individuals were only involved in updating the formularies during the review process as formularies were created at the outset of the scheme. The entire process of decision-making was based on the following sub-themes: 1. the flow of information in the medicines review process and 2. criteria for medicine selection and evidence-based medicine. These are described in this section together with other factors influencing the process of medicine selection.

#### The flow of information in the medicines review process

**Fig. 1:**
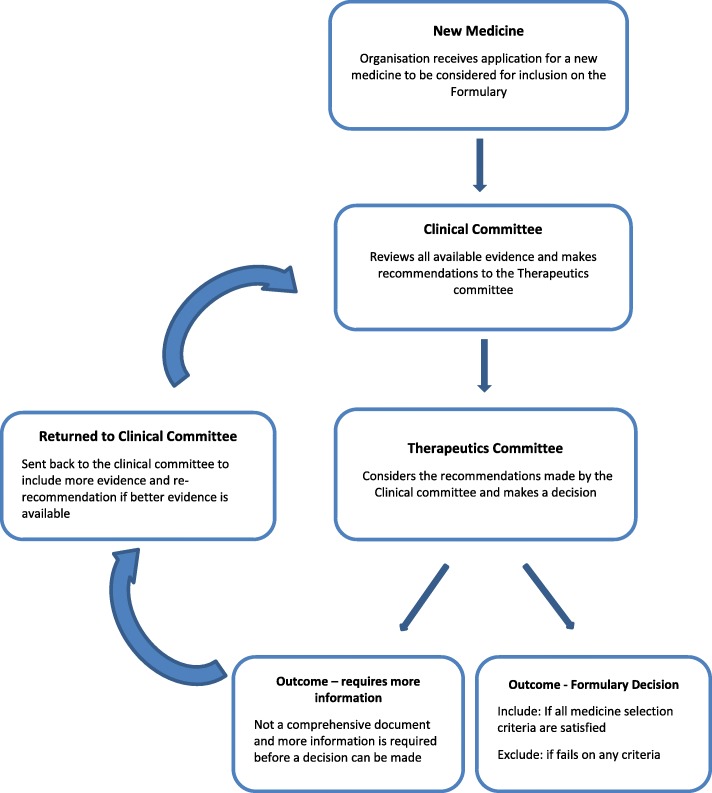
The flow of information for the medicines selection process

Generally, most companies performed a full review of all their formularies annually to ensure the formularies were still compliant with PMBs and CMS algorithms as there might have been additions and/or deletions of medicines to the various benefit options during the year on an ad-hoc basis when the need arose. In general, generic entities were simply added on if all criteria for generic inclusion were met but a new chemical entity or a new “me too” medicine was subjected to a review process. The following schematic of the flow of information in the medicines review process (Fig. 1) was developed from the information provided by the participants.

**Table 1:** Demographics of the study sample

Characteristics of sample	
**Gender**	2 - male
	8 - female
**Average age**	42 years
**Age Range**	31–54 years
**Profession**	2 - medical doctors
	8 - pharmacists
**Average number of years in formulary management**	10 years

#### Criteria for medicine selection and evidence based medicine

The process for medicine selection was similar. Generally, a new medicine was added if it was within a class or was a generic that did not cost more than what was already on the formulary. If it was a new chemical entity or a ‘me too’ it might have been subjected to a review process and was not automatically added. The various criteria used in the medicine selection process are described below.

##### a) Inclusion Criteria:

All schemes described the same basic criteria for a medicine to be considered for inclusion on a formulary viz. registration with the Medicines Control Council (MCC) (now known as the South African Health Products Regulatory Authority (SAHPRA)); clinical safety; efficacy; the correct registered indication reflected on the application for inclusion; performance with comparators; availability; cost-effectiveness; and implications for benefit design or if it fell under a PMB. Although these initial criteria for selection are very rigid other factors also play a role, such as the inclusion of generics; no review of generics deemed bioequivalent by MCC (SAHPRA) were conducted, these were added to the formulary. One participant commented as follows:

“The use of generics makes a huge contribution towards reducing expenditure related to medicines in our context. So we support them quite clearly and unashamedly really. As a result we are not really in a position to argue with or to doubt the validity of the MCC (SAHPRA) process”.

##### b) Exclusion Criteria.

Exclusion criteria for deciding to not add a medicine onto a formulary included: if the medicine was not registered; if it did not meet the minimum inclusion criteria of being safe, efficacious or was not a cost-effective option.

Reasons for deleting a medicine from the formulary included: if the medicine was withdrawn from the market, discontinued, if there were serious safety alerts, if an originator medicine became unaffordable when genericised, if a treatment guideline changed and the medicine was no longer on the algorithm then there was no obligation to pay for it as a PMB. A medicine might also automatically be removed from the formulary based on reference pricing. It would still be listed on the formulary but as a non-preferred item and would no longer be available for that specific benefit option.

##### c) Safety criteria.

Adequate safety and efficacy were fundamental to the acceptance of new medicines and were viewed in a serious light especially for a new chemical entity. Many companies did independent research on the safety of a medicine for inclusion on a formulary despite being approved and registered by the MCC (SAHPRA). This included looking at clinical trials data and package inserts provided by the manufacturer; a search for globally recognised sources for safety information such as FDA, NICE, CSM reports; post-marketing surveillance and pharmacovigilance studies.

One participant expressed concern with the availability of safety data for an extended period:

“it’s difficult to know what will happen prospectively and what happens after long term use, so we can only really take the information that we have available to us and that’s often in short term studies or relatively short term compared with the disease, so generally speaking we take the safety analyses outlined in the clinical trials but we do have a look for independent reviews and they often highlight negative effects not specifically observed or reported in the randomized controlled trials”

One company had a very comprehensive checklist for safety criteria:

“We have a checklist and we see what the company presents us with and what they present us with must correspond with what we have on our checklist … So we look at what is included in the drug evaluation document that is provided by the companies but it’s also nice to ask them the questions that aren’t always included in the document”.

To the contrary, one company felt that further safety evaluations were not necessary if the medicine was registered with MCC (SAHPRA) as this would imply all criteria were met.

##### d) Formulary Exclusions.

Some schemes had an out-right exclusion on vitamins (no vitamins were paid for) but those required for treating a condition were still covered:

“If they are not funding vitamins, they will still be funding your single vitamins and minerals where it is indicated for a specific chronic condition for e.g. they’ll cover your calciums and your potassiums, folic acid with methotrexate. But the combination multivitamins they sometimes have as an exclusion”.

##### e) Formulary exceptions.

All participants agreed that formulary exceptions applications/modifications were dealt with on a case by case basis. These were usually motivation-required medicines and defined clinical criteria were to be met. The procedure for consideration as an exception was explained as follows. A patient’s full clinical history was investigated, taking their utilization or claims pattern into account as well as their doses to check if the patient was on optimal doses of their current therapy. If they were on maximum doses and experienced side effects then an add-on therapy was considered. If the desired outcome was still not achieved, then it was looked at as an exception. There might also be an appeals process in place if an out of formulary medicine was required:

“If a person cannot use a specific in-formulary item then an alternative in-formulary item must be used. If this is still a problem then there is an appeals process. An appeals committee sits every week, sometimes twice a week for appeals, not just formularies but policy, when patients don’t meet their policy requirements and they want a drug that we won’t fund or for complicated cases, then formulary overrides can be done”.

“We will look at the motivation, what’s available on the formulary, what sort of non- formulary products is vital for the patient and doctor’s opinion. One will look definitely at the evidence … So, look at if it is clinically indicated and then we could authorise it … maybe there’ll be a non-formulary co-payment or if it’s been appropriately applied for, they went through all the protocol and all the steps it might even be there’s nothing on the formulary that works for this patient, we will waive the non-formulary co-payment for this”.

##### f) Off-label use.

The discussion surrounding medicines for off-label use was quite contentious. Respondents stated that schemes did not openly advocate off-label use of medicines and medicines were not included on a formulary specifically for off-label use. They recognized that this was a tricky and difficult situation to manage as there were numerous medicines used as standard of care that were off-label. They also argued that after a medicine was added to the formulary they were not always aware it was being used off-label. However, if there were no other options for the patient and if it was standard of care, or in the case of rare diseases there was some supporting evidence, a scheme would consider funding the medicine but were not obliged to do so.

“In the scheme rules it does say that the schemes don’t have to pay for a drug if it hasn’t been reviewed by the managed health care company, being us, for that indication. So it does cover them for the off label use”.

“in principal we don’t overtly support off-label use, so then we would need to have a look and see what have they used, so it would be sort of a case by case analysis, what has the patient used, has it been reasonable, what is the prevailing practice”.

##### g) Provision of information for medicine evaluation.

Participants stated that information for a medicine’s evaluation was provided by the pharmaceutical manufacturers. However, in every instance the schemes also performed their own literature searches for evidence, which remained the foundation for their decision-making. The information provided by pharmaceutical industry was useful and the respondents agreed that there was valuable information included such as the package inserts or clinical trial papers. However, it was commented that their pharmacoeconomic analyses/models and marketing material were viewed with great caution. Responses were as follows:

“So it starts off with initial foot-work or preparation of all the clinical information available … Wherever you got the South African diabetes guidelines, asthma guidelines or lipid guidelines, we take that into consideration as well… conditions where there’s no approved algorithm, we would then go into the EDL to see what is best practice prevailing in the public sector”.

“New chemical entities are subject to a formal submission process… So drug companies make the submission using that template and they submit the literature that they deem to be relevant. We then conduct our own internal literature review process … So to try and get a balanced view, between what they submit versus what we submit, and then obviously the external committee members have their own experience and their own clinical expertise that they bring to the discussion which enables us to make an informed decision”.

All participants stated that their decision-making was an evidence based process. Some comments included:

“We use the hierarchy of studies, the best evidence is obviously what you would hope for - good phase three studies, but depending on the scenario you would need to use case studies”.

“Yes they are evidence based. So randomised control trials, systematic reviews, meta-analysis are our preferred levels of evidence”.

##### h) Economic evaluations

Most companies did not do formal pharmacoeconomic analyses. In most cases this model was supplied by the pharmaceutical manufacturers but companies did predominantly do their own cost minimization evaluations. Responses were as follows:

“What is provided to us by the companies and what we usually insist on is the price comparison table. Then we look at comparisons of the submitted drugs and the comparators. We look at SEP, average daily use, average daily drug cost per patient, typical annual drug costs, range of plausible costs, ICERS, QALYs, DALYs if relevant. We look at sub-group analyses, discounting, discounting future outcomes. It’s also quite a protracted analysis. But we don’t analyse it ourselves, we expect the companies to provide models. We take what’s provided and see whether it’s relevant or not”.

One company had a very interesting viewpoint on pharmacoeconomic studies provided by manufacturers:

“I think that we have found that pharmacoeconomic studies are generally used inappropriately to justify high costs of medicines with limited clinical benefit … they tend to gloss-over some of the fundamental flaws that have come through in the efficacy and safety data. So we sort of beat the drum about safety and efficacy but once we’ve passed that step we generally follow cost minimisation principles”.

Another respondent said that it was important to look at the cost of care holistically:

“a unit price I’m paying for the more expensive one per unit holistically if you look into the total cost of care that now I don’t have a half day admission to hospital so what’s the bottom line for the scheme then, the more expensive one actually costs me less so you need to take into account the full circle, you can’t only stop at looking at per unit cost”.

##### i) Therapeutic interchange

All companies stressed that they did not do therapeutic interchanges. If an off-formulary item was requested (either due to the benefit option or was not listed on the formulary at all), the company then responded with the reasons for not being able to fund the medicine and might/might not have provided a list of in-formulary alternatives for the patient and physician to discuss. The patient was not denied the right to the medicine but was merely informed that a co-payment might be necessary.

##### j) Restrictions on use of medicines

Additional approval processes for formulary medicines was viewed as uncommon by all companies and was not something routinely done since all medicines on the formulary were available to the patients within their selected benefit option, provided they met the clinical entry criteria for a specific algorithm. An additional approval process, in most cases, was pertinent to a medicine not on the formulary or to the very high cost treatments. Responses were as follows:

“All biologics have to be approved by me (medical manager) and indications for those are usually rheumatological, ophthalmological. It is mainly a cost implication because it’s such a massive cost associated with the drugs”.

The use of some in-formulary medicines might have restrictions attached to them. Reasons for this were related to cost implications; or were only available with motivation from a specialist such as psychiatrists; or time period restrictions for treatment and follow up. Such scenarios are explained below:

“Your specialty medicines, the more expensive ones where we do have a protocol in place and you want to check the patient at least used this or that or complies to a specific clinical measure or test that’s being done. Time period to motivations - 48 hours to a week. If it’s an emergency situation, like a baby who needs an RSV immunization - within in a day”.

“in terms of restricting time periods … if it’s a drug that requires specific monitoring or it’s really expensive or it’s toxic or has limited benefit in conditions where there’s a large unmet need then we would potentially limit the duration of authorisation so that we’d able a follow up report to be received”.

#### Benefit design and reimbursement of medicines

Generally, company formularies were designed using different levels of reimbursement within the formularies. This allowed the companies to have for the same condition, different formularies providing a range of benefits to the members. On the high-end formularies there was more choice, whilst the low-end formulary was very restricted. Acute and chronic benefits might be included in the low-end options and a reference pricing methodology might be used in the more comprehensive, high-end formularies. This facilitated more choice to members but this methodology was used to manage the risk of cost for the company. The high-end formularies were described as “benefit-rich” and were the more expensive options. All companies used the SA EML (Essential Medicines Lists) in their benefit design process to ensure their formularies covered the essential medicines offered to a patient in the public sector for the PMBs.

It was evident from the interviews that after clinical safety and efficacy were determined; the cost of a medicine was then considered for its inclusion on a formulary and its position within the various benefit options. If a medicine met the initial criteria it might still not be added to a formulary if it was unaffordable or if added then it might have been restricted to the very comprehensive, expensive benefit options. The reimbursement system in all the companies referred to the use of a reference price methodology. One participant explained how this reference pricing affected a generic medicine for formulary inclusion:

“The moment a new one enters the market and it is cheaper than our reference price, then that will automatically be included… When your product comes in within our reference price or below, you are actually within formulary and that varies per benefit option”.

It was mentioned that the reference pricing methodology also indirectly addressed the issue of medicine shortages i.e. if a certain generic became unavailable due to stock shortages there were other generics in-formulary that a patient could have access to, although this might/might not incur a co-payment. In such an instance, manipulation of the reference price was possible to achieve this.

### Monitoring and evaluation of outcomes

Monitoring medicine use by patients was performed in each company in the sample but at different levels of intensity and in different ways. Some companies analysed their claims data and performed compliance monitoring whilst others had comprehensive disease management programmes to follow up on patients. In most companies, these processes were not regularly conducted for all patients but were mainly for the high cost medicines and/or patients.

“Regarding compliance monitoring, there’s a department that looks at high cost cases so they not looking at compliance for all people/patients”.

“Basically medicine utilisation trends are monitored on a quarterly basis where we assess cost per life per month. We assess chronic prevalence. We have a look at the top 10 most frequently used classes of drugs, products by cost, by frequency, we analyse those trends. So that’s done on a routine basis but then in terms of individual patients we have an active disease management programme which monitors selected high risks and potentially high cost patients … ”

“Compliance monitoring especially in high risk areas. We don’t routinely do outcomes data because it’s very time dependent and very deep analytics”.

Companies also performed safety monitoring of medicines and communicated this to their patients.

### Challenges

From the interview data an overarching theme describing challenges companies encountered with the medicines selection process and formulary management emerged. This is described in Table 2. which illustrates companies’ issues with (a) the provision of information for decision-making; (b) lack of health economic information; (c) alignment of formularies with SA EML; and (d) pressure from pharmaceutical industry.

**Table 2:** Participants’ opinions on challenges experienced with formulary development and management

Challenge	Comment
issues with provision of information for decision making	“one big issue … is the lack of update on the therapeutic algorithms, which is published by the council of medical schemes, Department of health and those”. “MCC (SAHPRA) … have failed … to indicate on a public list what products are generically equivalent”
Lack of health economic information	“You would go do a literature search to see if anything has been done, but what limits it in the South African market is that, you can’t really compare our economic situation and rand value to what’s been done in Europe and America. We do look at that but we try and consider the implications that the South African environment would have”. “We look into their opinion but we can also extrapolate that into some more international, local publications, if available you know, sometimes that’s of course the main restriction, is the non-availability of this type of pharmacoeconomic data”. “We don’t do the studies our self, but if somebody does submit a cost utility or cost effectiveness analysis, we would consider these. We don’t conduct them internally and they generally are viewed cautiously because it’s quite easy to manipulate the data to support their own needs. So, it’s sometimes quite difficult to interpret some of the costings they’ve used and some of the assumptions and they often base them on different economic settings which are different from a South African context”. “You hardly ever, ever, ever see pharmacoeconomic analysis that are endorsed provided in general literature from the pharma companies, what they do have is they bring individual studies that has been conducted in the world somewhere, when they want certain high cost drugs to be paid or when they want to compare products to each other. But it’s not always comprehensive enough to make a conclusive decision of whether it’s eligible for formulary listing or not”.
Alignment of formularies with SA EML	“It’s difficult to align EDL into normal daily business when EDL is not coded and integrated and they use international non propriety names to define the EDL list and if you don’t code your database with the international non-propriety names from the WHO, ATC classification, you won’t just automatically make that relationship with that. So coding, pharmaceutical coding is a huge part of integration, costing and projections”.
Pressure from pharmaceutical industry	“Sometimes you get companies saying: ‘can you then only pay for my products, if we reduce our price?’ and only pay for you and not pay for anybody else? We sorry, can’t do that, you know, we’ve got to be fair. It’s got to be open and transparent to everybody”.

## Discussion

### Main findings

The results from the interviews showed that all companies operated formularies with various benefit options. There was also a formal process for the selection of medicines for these formularies, be it a generic, a new chemical entity or a “me too” medicine. It was found that the process for a generic entering the market was quite simple as it might be added onto a formulary without a review process if all preliminary criteria were met. A full review process was applicable for new chemical entities and new “me too” medicines. Formularies were generally reviewed annually but might also be adjusted or updated many times during the year due to need. The primary reasons for updating included the addition or deletion of medicines due to price changes or safety warnings or new medicines entering the market or changes in treatment algorithms. The process of medicines selection was inherent in the review process since formularies were already in existence and were merely being updated.

The two most important committees involved in the medicine selection and review process were the Clinical team and the Therapeutics team. The Clinical team was responsible for all the background research and the team used evidence based medicine to compile a medicine evaluation document with all information, including those provided by the pharmaceutical industry, for the medicine in question. This document was then presented to the Therapeutics team for consideration. The multi-disciplinary representations from external expert consultants on the Therapeutics committees were fundamental to the decision making process as they not only brought their expert opinions to the floor but they maintained a degree of impartiality to the medicines selection process. A range of other units within a company might be consulted during the medicines selection process depending on the issue at hand. These units included: risk management, health economics; drug reimbursement, just to name a few.

All companies were compliant with and designed their formularies in accordance with the CMS PMBs and algorithms. Benefit design and medicine selection was a complex process given the number and nature of the various selection criteria and companies must be able to provide safe, effective medicines at affordable rates, within the PMBs framework. It is for these reasons companies abided by their formularies and were strict with medicines provision within benefit options. However, there were mechanisms in place (such as a motivation from a prescriber and an appeals process) for members who required medicines outside their benefit options, expensive medicines, or completely off-formulary items.

After a medicine satisfied the minimum criteria for safety and efficacy it was evaluated on cost. Cost was an important factor in the decision to include a medicine at a specific benefit option. Companies limited spending by employing medicine formularies; employing a medicines reference pricing tool, and with the use of generics. Other cost-containment techniques used by companies included: (i) established-use criteria: where a patient must have satisfied all the criteria and followed all the protocols and failed on all the algorithms to eventually qualify for a requested medicine that was outside their benefit option, (ii) restricted use: limiting use of certain medicines to specially trained individuals such as ensuring specialist initiation and follow up for certain conditions that required monitoring; or limiting periods of authorisation for medicines requiring follow up evaluations. Companies also monitored use of medicines by patients, from claims data analyses to compliance monitoring, which enabled patient profiling for cost estimates for that patient and healthcare in general for managing costs for specific diseases. Monitoring of use of medicines by patients was done in all companies but varied in degree and type. Thus, for some companies, there remained room for strengthening, by expanding the range of monitoring methods and techniques used.

Formulary managers perceived the lack of information as hindering the formulary management process. The first of these was stated to be the lack of availability of therapeutic algorithms and a list of approved generic equivalents. There seemed to be some confusion amongst formulary managers as to the roles and responsibilities of the NEMLC on the one hand and the CMS on the other. Going forward it would be useful for the National Department of Health to clarify and inform stakeholders as to the roles and responsibilities of these two organisation and the future NHI list for reimbursement committee. Secondly, formulary managers expressed their concern with lack of appropriate pharmacoeconomic information and studies pertinent to SA. It is recommended that rigorous pharmacoeconomic evaluations be included in some cases when reviewing medicines, especially new medicines. Such evaluations should include considerations for all costs and consequences pertaining to the use of that medicine. In the case of reviewing a generic equivalent, cost-minimisation may be used [15]. It was found that all companies in the sample used cost-minimisation. American Society of Health-System Pharmacists guidelines (2008) suggested that decision analysis models incorporating local data can be employed when published pharmacoeconomic data is lacking [15]. However, it is not known if companies did employ this recommendation as it did not form part of the scope of the study.

### Comparisons with other organisations’ formulary processes

The formulary processes described in this study were similar to those of CVS/caremark, which is the prescription benefit management subsidiary of CVS Health in the United States of America. This company has openly published their formulary management process [16]. Their underlying principles for the formulary development and management processes included: (a) the provision of a clinically appropriate formulary: (b) formulary decisions made by a Pharmacy and Therapeutics committee, the membership of which comprised independent, unaffiliated clinical pharmacists and physicians. There is also a Pharmacy and Therapeutics subcommittee in place, the membership of which comprised members from the CVS/caremark clinical departments. The subcommittee met monthly to review new FDA approved medicines entering the market and provided recommendations to the national Pharmacy and Therapeutics committee. Together these committees managed the formulary process supported by (i) the clinical formulary department, who were responsible for the provision of information, medicine monographs and Therapeutic class review and; (ii) Formulary review committee who evaluated additional factors affecting the formulary such as utilisation trends; the impact of generic drugs; and (c) left the ultimate prescribing decision and therapeutic plan to the prescriber. Formulary decisions were reported to be evidence based and considered standards of practice, accepted clinical practice guidelines as is also reportedly done in our SA study. All formularies are reviewed annually by medicine class to ensure previous recommendations were maintained and to recommend additional changes to maintain clinical appropriateness based on new information. Formularies were designed on tiered co-payments, which promoted the use of preferred formulary products; a closed formulary option was also available which covered a set number of products only, unless a claim went through an override process. They also promoted the use of generics in the formulary, as is also done in our SA study, as well as have a member-directed formulary education component which communicated medicine information to members.

The Academy of Managed Care Pharmacy has also published information on its formulary management [17]. A Pharmacy and Therapeutics committee was responsible for the development, management, maintenance and administration of the formulary. Membership of the committee included primary healthcare and specialty physicians, pharmacists; nurses; legal experts and administrators. Members were independent to the benefit organisation and declared conflicts of interest. The committee also designed and implemented formulary system policies on use and access to medicines. Medicine utilization strategies included quantity limits, step therapy and prior authorisation criteria for consideration by the committee. Policies on access to medicines included an exception process to allow use of a non-formulary medicine under clearly defined circumstances. The membership of this team was slightly different to that reported in our SA study, where the therapeutics team may comprise members from the medical scheme but the policies on access to medicines for non-formulary items aligned well with the SA process for items requiring a motivation.

Express scripts’ National Preferred formulary is the most used medicine list in the United States and provided medicines coverage for 25 million people [18]. Their principles for formulary development included: (i) clinical appropriateness of the medicine before cost; (ii) the physician made the final decision for the patient’s treatment; (iii) the use of evaluations from autonomous physicians. Their formulary development was guided by (a) the National Pharmacy and Therapeutics Committee (consisted physicians and pharmacist); (b) Therapeutic assessment committee (internal clinical review team comprised physicians and clinical pharmacists employed by the company who were responsible for the evidence based review of medicines); and (c) Value assessment committee (comprised company employees from formulary management, product management, finance and clinical account management, who were responsible for assessing the value of medicines). Formulary decisions were as follows: (a) included medicines that were safe, effective, for indicated use, and demonstrated clinical benefit not provided by any other medicine on the market; (b) excluded medicines where safety risks outweighed clinical benefits; (c) medicines were considered optional, where it is safe, effective for indicated use and might be included on the formulary. Such medicines usually had other clinical-equivalent alternatives. The principles for formulary development in SA compared well with these described for Express scripts as clinical appropriateness (safety and efficacy) was considered first before cost for formulary inclusion and the physician made treatment decisions for patients in line with the benefit packages as offered by the medical schemes. Values assessments were also done in SA but were not as clearly defined.

### The importance of the formulary process

Apart from the production and maintenance of a formulary, a formulary system included the methods employed by the organisation to evaluate evidence and their approach for medicines selection for various diseases, medical conditions and patients. An organisation’s policies and procedures for medicine procurement, authorisations, and appropriate use of medications by patients were also included in a formulary system. Often a formulary system might have additional clinical guidelines and information for prescribers and other healthcare professionals, for the provision of quality, affordable care for patients. Furthermore, formularies have policies that allow physicians and patients the opportunity to access non-formulary medicines when required - this ensures quality assurance [17].

Formulary decisions impact on all aspects of healthcare management. The increasing number of medicines, production of complex medicines, and escalating medicine costs makes medicines selection a necessary but complex task. The formulary management process afforded medical schemes the ability to distinguish between superior and marginal medicines. This efficient and effective use of healthcare resources potentially reduces overall healthcare costs; enhances patient access to more affordable care thereby contributing to an improved quality of life [17].

The participants of our study successfully unpacked and described the decision-making policies and processes for formulary development and management and it is evident that the medical schemes in SA have all the components of a successful formulary system, as described above, and contributes to improved access to medicines for private sector beneficiaries.

### What can be learned from the private sector medical schemes?

The move towards NHI is unnerving for private sector medical schemes as it poses serious ramifications. The NHI Bill passed in August 2019, stated that medical schemes will only be allowed to fund complimentary cover for services not offered by the NHI package of benefits and medicines approved by the NHI benefits advisory committee. The number of medical schemes is expected to plummet. It is therefore important for medical schemes to align themselves with the NHI roadmap. The NHI Bill also stated that a key element of improving service delivery is to ensure that the full range of essential medicines and other medical supplies are available in all public health facilities [19]. The WHO Lancet commission believes that UHC may be achieved by policies that support essential medicines which must support health services delivered through mixed systems including both the public and private sectors [8]. A recent study conducted by Perumal-Pillay and Suleman (2017) showed that the monitoring and evaluation component of the Essential medicines programme and EML by SA National Essential Medicines List committee required strengthening. It was shown that evaluation of their policy decisions and monitoring use of medicines by patients required attention to strengthen the country’s EML programme and policy [20]. From our current study, it is evident that these very aspects that are wanting in the public sector EML management processes are addressed in the private sector medical scheme formulary management processes. Furthermore, from the interviews it was clear that many medical scheme companies were concerned with the total cost of care of managing a disease in a patient and not just the cost of the medicine on the formulary. This was important to prevent downstream costs with that patient. Patients were monitored and interventions applied when needed to improve patient care, rational use of medicines and improved patient outcomes, which all ultimately impact on costs incurred with management of that patient and disease. These concepts were fundamental in the disease management approach, which is patient-focused. Although the future of the private sector is uncertain under the new NHI, much can be learned from them in this regard. Knowledge sharing of the medical schemes’ policies and procedures surrounding the provision of medicines, including essential medicines, may be one way of providing supporting information to and aligning with the NHI system. This study contributed to transparency with medicines policies in the country as it placed this information in the public domain for use by policy makers concerned with medicines policies both nationally and internationally.

### Strengths and limitations

The study gathered perspectives and opinions from formulary managers from private sector medical schemes and administrators about their experiences with formulary development and management and the committees responsible for the decision-making process. The sample included medical schemes and administrators that represented the majority of the insured population as it included 3 schemes and administrators that insured more than three quarters of the total membership of schemes, at the time of the study. However, valuable opinions from the medical schemes, administrators and managed care organisations that insured the remaining quarter of beneficiaries were not captured in the study. Familiarity with medicine selection processes may have been over-reported as participants were provided the opportunity to peruse the interview questions prior to the interview.

### What this study adds and suggestions for future studies

This research has brought to the fore, a collated response on the process of medicines selection in the private sector and the policies surrounding it. It has captured the observations, experiences, and opinions of formulary managers from medical schemes, medical scheme administrators and managed care organisations. This is one of the first research studies in SA to publish the process of private sector medicines selection in the country improving transparency in the process. More research is required into the preauthorization process and the use of Designated Service Providers and how this impacts the formulary. The impact of these policies employed in the formulary management system can be measured by gathering perspectives from patients accessing healthcare through the private sector medical schemes. Further studies are needed to confirm this.

## Conclusions

This is one of the first studies in SA to report on how decisions are taken to include or exclude medicines on formularies of private sector medical schemes, administrators and managed care organisations. It provides an insight into their medicine selection, review and monitoring policies and processes employed. The study reveals that formulary development and management is a complex, multifaceted process. The lessons learnt from the SA experience with private sector formularies, and medicine selection is a useful comparison for other countries that have existing public-private sector differentiations as they move towards universal healthcare. It is important to understand that values may be different in the sectors and these need to be aligned so as to develop mutually accepted benefits packages.

## Data Availability

The data supporting the conclusions in this manuscript are included within the manuscript in the participants’ verbatim quotes, tables and figure.

## Abbreviations

CMS: Council for Medical Schemes
EDL: Essential Drugs List
EML: Essential Medicines List
MCC: Medicines Control Council
NHI: National Health Insurance
PMBs: Prescribed Minimum Benefits
SA: South Africa
SAHPRA: South African Health Products Regulatory Authority
UHC: Universal Health Coverage
WHO: World health organization
